# Proteomic Analysis Revealed the Characteristics of Key Proteins Involved in the Regulation of Inflammatory Response, Leukocyte Transendothelial Migration, Phagocytosis, and Immune Process during Early Lung Blast Injury

**DOI:** 10.1155/2021/8899274

**Published:** 2021-04-27

**Authors:** Yunen Liu, Changci Tong, Peifang Cong, Ying Liu, Xiuyun Shi, Lin Shi, Shun Mao, Yan Zhao, Hongxu Jin, Mingxiao Hou

**Affiliations:** ^1^Shenyang Medical College, No. 146, Huanghe North Street, Shenyang 110034, China; ^2^The Second Affiliated Hospital of Shenyang Medical College, The Veterans General Hospital of Liaoning Province, No. 20 Beijiu Road, Heping District, Shenyang 110001, China; ^3^Jihua Laboratory, No. 28 Island Ring South Road, Guicheng Street, Foshan City, Guangdong 528200, China; ^4^Department of Emergency Medicine, General Hospital of Northern Theater Command, Laboratory of Rescue Center of Severe Trauma PLA, No. 83 Road, Shenhe District, Shenyang l10016, China

## Abstract

Previous studies found that blast injury caused a significant increased expression of interleukin-1, IL-6, and tumor necrosis factor, a significant decrease in the expression of IL-10, an increase in Evans blue leakage, and a significant increase in inflammatory cell infiltration in the lungs. However, the molecular characteristics of lung injury at different time points after blast exposure have not yet been reported. Therefore, in this study, tandem mass spectrometry (TMT) quantitative proteomics and bioinformatics analysis were used for the first time to gain a deeper understanding of the molecular mechanism of lung blast injury at different time points. Forty-eight male C57BL/6 mice were randomly divided into six groups: control, 12 h, 24 h, 48 h, 72 h, and 1 w after low-intensity blast exposure. TMT quantitative proteomics and bioinformatics analysis were performed to analyze protein expression profiling in the lungs from control and blast-exposed mice, and differential protein expression was verified by Western blotting. The results demonstrated that blast exposure induced severe lung injury, leukocyte infiltration, and the production of inflammatory factors in mice. After analyzing the expression changes in global proteins and inflammation-related proteomes after blast exposure, the results showed that a total of 6861 global proteins and 608 differentially expressed proteins were identified, of which 215, 128, 187, 232, and 65 proteins were identified at 12 h, 24 h, 48 h, 72 h, and 1 week after blast exposure, respectively. Moreover, blast exposure-induced 177 differentially expressed proteins were associated with inflammatory responses, which were enriched in the inflammatory response regulation, leukocyte transendothelial migration, phagocytosis, and immune response. Therefore, blast exposure may induce early inflammatory response of lung tissue by regulating the expression of key proteins in the inflammatory process, suggesting that early inflammatory response may be the initiating factor of lung blast injury. These data can provide potential therapeutic candidates or approaches for the development of future treatment of lung blast injury.

## 1. Introduction

Blast injury is a special type of trauma caused by shock wave overpressure that directly or indirectly acts on the body. In recent years, blast injuries have become an increasingly serious problem in military and civilian practice and have had a huge impact on all regions of the world. For example, explosion accidents in Tianjin port on August 12, 2015 [1], and in Taiwan on June 27, 2015 [2, 3], were caused by chemical dangerous goods, dust explosions, and gas explosions in factories. These blast injuries not only caused economic damage but also seriously affected people's life. In Western countries, terrorist attacks (such as these in Paris in 2015 and Sri Lanka in 2019) and local conflicts (such as the wars in Afghanistan and Iraq) have also caused one out of 10 military casualties and 6-9 out of 10 civilian victims suffered blast injuries [4–6].

A large amount of in vivo and in vitro data showed that the lungs, brain, gut, ears, and sinuses are susceptible to high-intensity blast wave. Among them, the impact on the lungs is greater, because the alveoli are filled with a single layer of epithelial cells and delicate vascular structures [7, 8]. Compared with the long-term consequences of brain blast injury, the early symptoms of lung blast injury are more serious and are the most important causes of casualties. Unlike other traumatic lung injury, lung blast injury usually does not produce obvious wounds on body surface, and its early symptoms and signs are not obvious, which is characterized by “light outside and heavy inside, rapid development.” When the chest is directly exposed to shock wave overpressure, the pressure will spread through chest wall tissue to the body and cause rupture of alveolar capillaries, intrapulmonary hemorrhage, and edema. Subsequently, intrapulmonary hemorrhage further causes the formation of free radicals, edema, infiltration of inflammatory factors, and oxidative damage, which may develop into acute respiratory distress syndrome [9–13]. Our previous studies found that blast injury caused a significant increased expression of interleukin-1 (IL-1*β*), IL-6, and tumor necrosis factor (TNF-*α*), a significant decreased expression of IL-10, an increase in Evans blue leakage, and a significant increase in inflammatory cell infiltration [10, 14, 15]. Moreover, we have previously reported that blast exposure-induced inflammation in the lungs occurred within the first 12-48 h and peaked at approximately 24 h [16]. Smith and Garner [17] reported similar results that inflammation affected the lungs, which developed from 24 h to 48 h and peaked at about 48 h. However, current treatment plan for patients with lung blast injury mainly focuses on supportive care, intensive therapy, and mechanical ventilation, and there is no specific alternative therapy. Therefore, in order to provide more other therapeutic approaches, clarifying the key target proteins of early inflammation of lung blast injury will be helpful for effective protection, treatment, and the development of new target drug.

Mass spectrometry- (MS-) based proteomics is becoming a widely effective method for identifying, characterizing, and quantifying proteins that are part of life's essential processes. MS-based proteomics are increasingly helping us understand the dynamics, interactions, and effects of proteins and peptides and improve our systematic understanding of biology [6, 18, 19]. A previous study used tandem mass tag (TMT) labeling quantitative proteomics technology to analyze differentially expressed proteins in the lungs of mice with or without exposure to fine particulate matter (PM2.5) and found that the extracellular matrix- (ECM-) receptor interaction, phagosome, and phosphatidylinositol 3-kinase- (PI3K-) protein kinase B (Akt) signaling pathways contribute to PM2.5-induced pulmonary fibrosis [20]. In addition, Sakaue et al. [21] used a proteomic approach to find that mRNA and protein levels of S100A9 and ANXA1 were upregulated in the lungs of mice with common bile duct ligation. Another iTRAQ LC-MS/MS analysis of differentially expressed proteins in bronchoalveolar lavage fluid from 36 patients with ARDS (20 survivors, 16 nonsurvivors) revealed that several signaling pathways implicated in lung injury and repair, including coagulation/thrombosis, acute-phase response signaling, and complement activation [22]. However, the molecular characteristics of lung injury at different time points after blast exposure have not been reported. In this study, TMT combined with liquid chromatography tandem mass spectrometry (LC-MS/MS) and Western blotting was used for the first time to investigate the global proteomics of lung blast injury at different time points.

## 2. Materials and Methods

### 2.1. Animals and Experimental Groups

Forty-eight male C57BL/6 mice (20-25 g, 6-8 weeks old) were obtained from the Experimental Animal Department of the General Hospital of Northern Theater Command. After acclimating for one week, all mice were randomly divided into six groups (*n* = 8/group): control, 12 h, 24 h, 48 h, 72 h, and 1 w after low-intensity blast exposure. All mice were kept in a room, maintained at a temperature of 20 ± 2°C and humidity of 55%–65%, and given unrestricted access to food and water. Animal welfare and experimental design were approved by the Ethics Committee of the General Hospital of Northern Theater Command.

### 2.2. Blast Exposure Induced Lung Injury

A precise model of lung blast injury was used as previously described [10]. Briefly, mice were anesthetized by abdominally injecting 2% pentobarbital sodium (1.5 ml/kg). After stabilizing the device with screws, mice were placed on rubber pads with 10 regular holes atop the device. The pressure pump was connected to the bottom of the device and was continuously pressurized until 5 layers of 18 mm thick aluminum film bursts. The compressed air rapidly expanded from the blasting port at high speed, forming shock waves that impacted the chest of the mice. The pressure detected by a pressure sensor was transmitted through a data cable and recorded by a computer. The mice after detonation fall into the prepared soft woven bag to avoid secondary impact damage. The overpressure value of the shock wave at the instant of blasting was 115.8 ± 10.4 per square inch (PSI). The mice in control group underwent identical procedures as blast groups only without blast exposure. After blast exposure, mice were removed from the woven bag and returned to the original cag.

### 2.3. Samples Collection and Pathological Examine

All mice were intraperitoneally anesthetized with 2% sodium pentobarbital (1.5 ml/kg), and lung samples were collected 12 h, 24 h, 48 h, 72 h, and 1 w after blast exposure, respectively. Vascular permeability was examined as described previously [23]. Briefly, 30 m before the mice were executed, Evans blue (10 mg/kg) was administered intravenously (i.v.; 1 ml/kg) via the tail vein. After blast exposure, a segment of the lungs was removed and dried in a culture dish for 24 h at 37°C. The dry weight was calculated, and Evans blue was extracted using 1 ml formamide for 24 h at room temperature. The amount of Evans blue in the lungs was quantified by measuring the absorbance at 620 nm in an ELISA plate reader (Imark, Bio-Rad, USA). The concentrations of the dye were calculated from a standard curve. The results are presented as the amount of Evans blue in mg/g of tissue. The whole lung of 3 mice was used for proteomics, the left lung of 5 mice was used for histopathological observation, and the right lung was used for Western blot analysis. For the histological analysis, lung samples were fixed in 10% formaldehyde at room temperature, processed, and embedded in paraffin blocks using a Leica Microsystem tissue processor (ASP 300S, Germany). For histological staining, 4 *μ*m thick sections were sliced using a Leica Microsystem microtome (Model RM 2265, Germany), which were stained with hematoxylin and eosin (H&E).

### 2.4. Protein Extraction

Lung sample was ground by liquid nitrogen into cell powder and then transferred to a 5 ml centrifuge tube. After that, four volumes of lysis buffer (8 M urea, 1% protease inhibitor cocktail) were added to the cell powder, followed by sonication three times on ice using a high-intensity ultrasonic processor (Scientz). The remaining debris was removed by centrifugation at 12,000 g at 4°C for 10 min. Finally, the supernatant was collected, and the protein concentration was determined with the BCA kit according to the manufacturer's instructions.

### 2.5. Trypsin Digestion

For digestion, the protein solution was reduced with 5 mM dithiothreitol for 30 min at 56°C and alkylated with 11 mM iodoacetamide for 15 min at room temperature in darkness. The protein sample was then diluted by adding 100 mM TEAB to urea concentration less than 2 M. Finally, trypsin was added at 1 : 50 trypsin-to-protein mass ratio for the first digestion overnight and 1 : 100 trypsin-to-protein mass ratio for a second 4 h-digestion.

### 2.6. TMT/iTRAQ Labeling

After trypsin digestion, peptide was desalted by Strata X C18 SPE column (Phenomenex) and vacuum-dried. Peptide was reconstituted in 0.5 M TEAB and processed according to the manufacturer's protocol for TMT kit/iTRAQ kit. Briefly, one unit of TMT/iTRAQ reagent was thawed and reconstituted in acetonitrile. Peptide mixtures were then incubated for 2 h at room temperature and pooled, desalted, and dried by vacuum centrifugation.

### 2.7. HPLC Fractionation

The tryptic peptides were fractionated into fractions by high-pH reverse-phase HPLC using Agilent 300Extend C18 column (5 *μ*m particles, 4.6 mm ID, 250 mm length). Briefly, peptides were first separated with a gradient of 8% to 32% acetonitrile (pH 9.0) over 60 min into 60 fractions. Then, peptides were combined into 18 fractions and dried by vacuum centrifuging.

### 2.8. LC-MS/MS Analysis

The tryptic peptides were dissolved in 0.1% formic acid (solvent A), directly loaded onto a home-made reversed-phase analytical column (15 cm length, 75 *μ*m i.d.). The gradient was comprised of an increase from 6% to 23% solvent B (0.1% formic acid in 98% acetonitrile) over 26 min, 23% to 35% in 8 min and climbing to 80% in 3 min then holding at 80% for the last 3 min, all at a constant flow rate of 400 nl/min on an EASY-nLC 1000 UPLC system. The peptides were subjected to NSI source followed by tandem mass spectrometry (MS/MS) in Q ExactiveTM Plus (Thermo) coupled online to the UPLC. The electrospray voltage applied was 2.0 kV. The m/z scan range was 350 to 1800 for full scan, and intact peptides were detected in the Orbitrap at a resolution of 70,000. Peptides were then selected for MS/MS using NCE setting as 28, and the fragments were detected in the Orbitrap at a resolution of 17,500. A data-dependent procedure alternated between one MS scan followed by 20 MS/MS scans with 15.0 s dynamic exclusion. Automatic gain control (AGC) was set at 5E4. Fixed first mass was set as 100 m/z.

### 2.9. Database Search

All MS raw files from the same batch were processed together with MaxQant (ver. 1.5.8) against the SwissProt *Mus musculus* protein database (version 2018.08, 16,992 entries), concatenated with the reverse decoy database. Trypsin/P was specified as a cleavage enzyme allowing up to 2 missing cleavages, 5 modifications per peptide. The mass tolerance for precursor ions was set as 20 ppm in first search and 5 ppm in main search, and the mass tolerance for fragment ions was set as 0.02 Da. The mass error was set to 20 ppm and 0.02 Da for precursor ions fragment ions, respectively. Carbamidomethylation on Cys was specified as fixed modification and oxidation on Met, and acetylation on protein N-terminal was specified as variable modifications. The minimal peptide length was set as 7 residues. The false discovery rate (FDR) of peptide and protein was all set as 1%.

### 2.10. Quantification of Global Proteome Data

The quantification analysis was performed at the protein level by MaxQuant software. TMT reporter ion intensities of each peptide were normalized by average in all samples. Protein quantitation was calculated from the median ratio of protein corresponding unique peptides when there were at least two unique peptides in a protein. Protein quantitation values were normalized by column-median to correct for equal loading across samples and then log2-transformed. All normalization steps were performed in RStudio.

### 2.11. Differentially Expressed Protein Analysis

Student's *t* test was used to examine whether proteins were differentially expressed between any two different group samples. Upregulated or downregulated proteins were defined as differentially expressed protein (DEP) in test compared control (ratio > 1.2 or ratio < 1/1.2, Student's *t* test nominal *p* < 0.05). Volcano plot of differentially expressed proteins was plot by the visualization R package “ggplot2.” All calculation and visualization steps were performed in RStudio.

### 2.12. GO Classification

Gene Ontology (GO) annotation proteome was derived from the UniProt-GOA database (http://www.ebi.ac.uk/GOA/). Firstly, DEPs were mapped to GO IDs by protein accession. If some DEPs were not annotated by UniProt-GOA database, the InterProScan soft would be used to annotate protein's GO functional based on the protein sequence alignment method. Then, DEPs were classified by Gene Ontology annotation based on three categories: biological process, cellular component, and molecular function. A bar plot graph was used to present GO terms by the visualization R package “ggplot2” in RStudio.

### 2.13. KEGG Pathway Enrichment

KEGG database was used to annotate protein pathway. Firstly, the KEGG online service tool KAAS was used to annotate protein's KEGG database description. Then, the result on the KEGG pathway database was annotated using the KEGG online service tool KEGG mapper. DEP enriched pathways were identified by a two-tailed Fisher's exact test. The pathway with *p* value < 0.05 was considered significant. A bubble plot graph was used to present enriched pathway by the visualization R package “ggplot2.” All calculation and visualization steps were performed in RStudio.

### 2.14. Protein-Protein Interaction Network

All DEP accessions were searched against the STRING database version 11.0 for protein-protein interactions. Only interactions between the proteins belonging to the searched data set were selected, thereby excluding external candidates. STRING defines a metric called “confidence score” to define interaction confidence; we fetched all interactions that had a confidence score > 0.7 (high confidence). Interaction network form STRING was visualized in the CytoScape software.

### 2.15. Western Blotting

Western blotting was performed as described previously [24]. Lung tissues were lysed in complete RIPA buffer (10 mM Tris-HCl pH 7.4, 150 mM NaCl, 1% NP40, 0.1% sodium dodecyl sulfate (SDS), 1 mM phenylmethylsulfonyl fluoride (PMSF), and 1× protease inhibitor cocktail (Roche)) and homogenized using a Sonic Dismembrator 100 (Fisher Scientific). The protein concentration of the tissue homogenates was measured using a Bio-Rad Protein Assay, and equal amounts of soluble protein were separated on 10% polyacrylamide gels, transferred onto a nitrocellulose membrane, and followed by routine western blot analysis. Primary antibody is as follows: TNF-*α* (1 : 2000, ab8348, Abcam, UK), IL-10 (1 : 2000, ab9969, Abcam, UK), IL-1*β* (1 : 2000, sc-7884, Santa Cruz Biotechnology, Inc., USA), TGF*β*1, Ahsg (1 : 1000, ab187051, Abcam, UK), Sema7a (1 : 1000, ab23578, Abcam, UK), Scgb1 (1 : 1000, ab40873, Abcam, UK), CD11a (1 : 1000, ab186873, Abcam, UK), Rac2 (1 : 1000, ab2244, Abcam, UK), PKC*α* (1 : 1000, ab32376, Abcam, UK), Mpo (1 : 1000, ab208670, Abcam, UK), Ncf1 (1 : 1000, ab795, Abcam, UK), H2-Ab1(1 : 1000, ab63567, Abcam, UK), Calr (1 : 1000, ab2907, Abcam, UK), Lbp (1 : 1000, ab233524, Abcam, UK), Hsp90ab1 (1 : 1000, ab53497, Abcam, UK), CD74 (1 : 1000, ab202844, Abcam, UK), Ltf (1 : 1000, ab10110, Abcam, UK), and Ankrd17 (1 : 1000, ab85726, Abcam, UK). Secondary antibody was goat anti-mouse secondary antibody (HRP) (1 : 4000 mouse IgG, ab6789, Abcam, UK), goat anti-rabbit secondary antibody (HRP) (1 : 4000, ab6721, Abcam, UK), and goat anti-rat secondary antibody (HRP) (1 : 2000, ab7097, Abcam, UK). Proteins were visualized using a ClarityTM Western ECL Substrate (170-5061; Bio-Rad Laboratories, Inc., USA) and a Tanon 5200 full automatic chemiluminescence image analysis system (Tanon Science and Technology Co., Ltd., Shanghai, China).

### 2.16. Statistical Analysis

Statistical analysis was performed using the SPSS 20.0 statistical software (IBM Corp., Armonk, NY, USA). All data were expressed as means ± SEM. Statistical comparisons were made by Student's *t* test for two groups and a one-way ANOVA test followed by Tukey's test for multiple comparisons. Differences were considered significant at *p* < 0.05 for all analyses.

## 3. Results

### 3.1. Blast Exposure Caused Severe Lung Injury, Leukocyte Infiltration, and the Production of Inflammatory Factors in Mice

**Figure 1:**
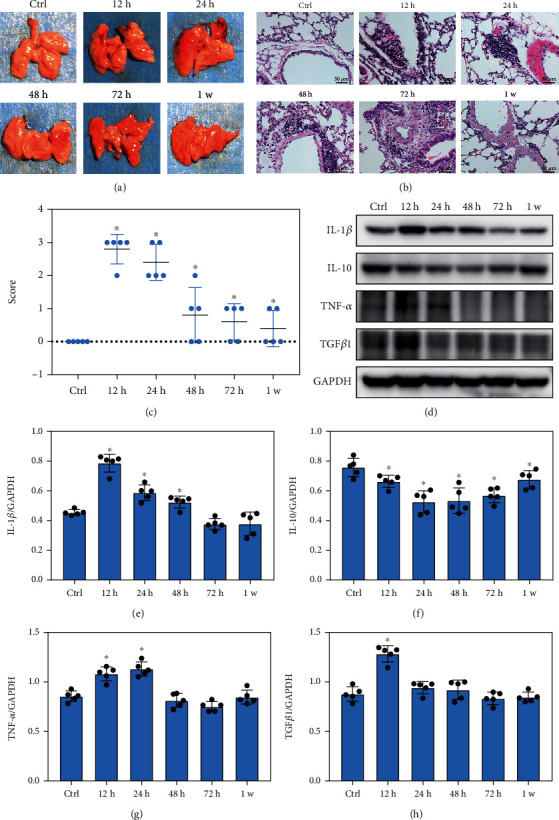
Blast exposure caused severe lung injury, leukocyte infiltration, and the production of inflammatory factors in mice. (a, b) Pathological changes of acute lung injury caused by blast exposure at different time points. (c) The score of pathological changes of acute lung injury. (d–h) Representative images of Western blotting analysis and quantitative analysis of IL-1*β*, TNF-*α*, TGF*β*1, and IL-10 expression in blast exposure-induced acute lung injury. All experiments were repeated at least three times. All data were expressed as mean ± SEM (*n* = 5) and analyzed by the one-way ANOVA test followed by Tukey's test for multiple comparisons. Differences were considered statistically significant at *p* < 0.05. ^∗^*p* < 0.05 vs. the control (Ctrl) group.

The explosion is thought to cause lung injury and increase myeloid cell infiltration in the lungs by promoting the expression of inflammatory factor. Our data revealed that blast exposure caused obvious hemorrhage, edema, and inflammatory cell infiltration in the lungs of mice (Figures 1(a)–1(c)). Compared with the control group, the expression levels of IL-1*β* and TNF-*α* in lung tissues were significantly increased at 12 h and 24 h after blast exposure and then returned to normal levels. Moreover, the TGF*β*1 level increased significantly 12 h after the blast exposure and then gradually decreased to normal level. In contrast, the expression level of IL-10 in the blast group was significantly reduced than that in the control group from 12 h to 1 week after detonation (Figures 1(d)–1(h), *p* < 0.05). These data indicated that blast exposure induced severe lung injury, lung leukocyte infiltration, and production of inflammatory factors in mice.

### 3.2. Differential Expression Profiles and Functional Annotation of Proteins in Blast Exposure-Induced Lung Injury to Different Time

**Figure 2:**
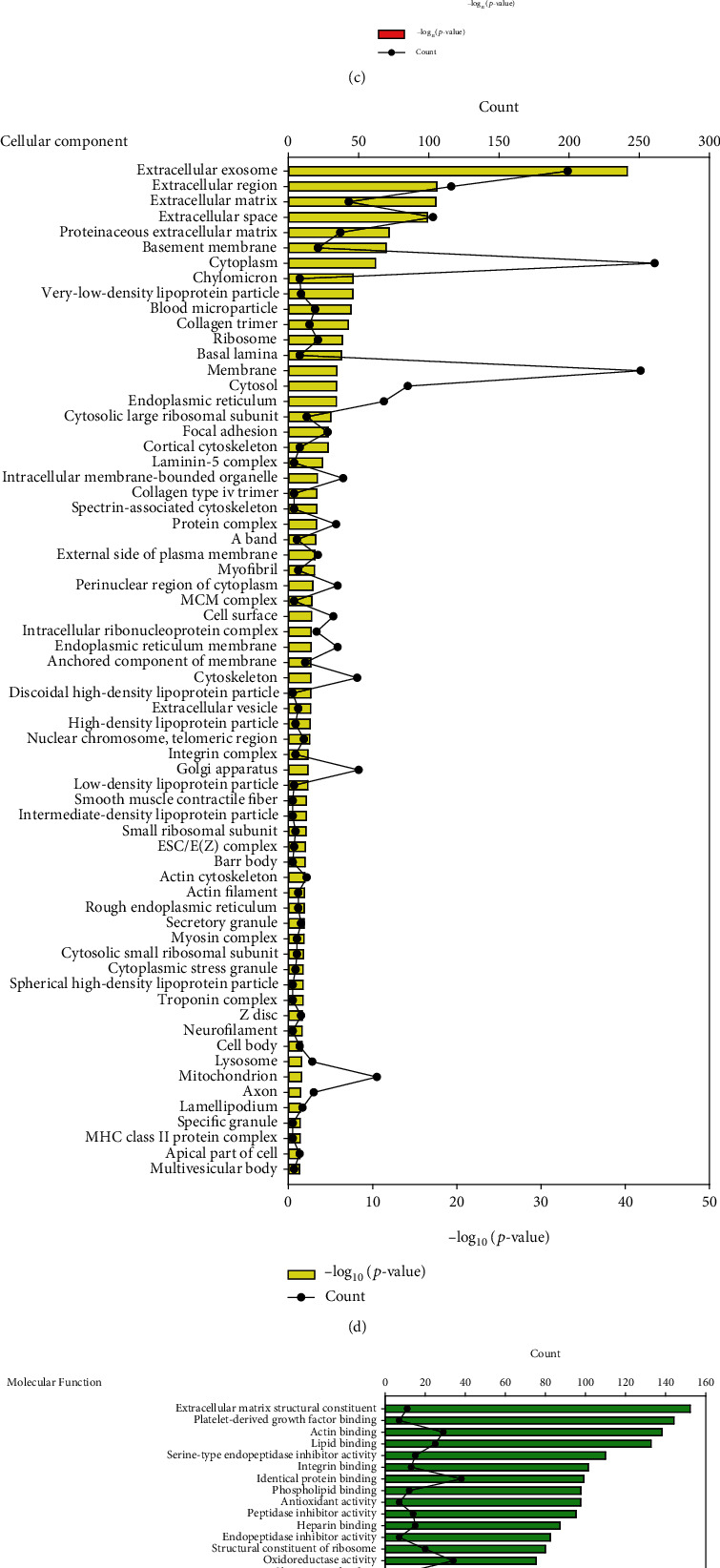
Global proteomics analyses and the identification of differentially expressed proteins in blast exposure-induced lung injury at different time points. (a) Hierarchical clustering heatmap of the differentially expressed proteins in the lungs on at different time points after blast exposure. (b) Venn diagram showing the distribution of differentially expressed proteins among groups. Overlapping parts represented proteins that appear at different time points. (c–e) GO analysis of differentially expressed proteins in the lungs at different time points after blast exposure. (c) Biological process. (d) Cellular components. (e) Molecular functions. (f) KEGG analysis of differentially expressed proteins of mouse lungs at different time points after blast exposure. (g) Network analysis of all obtained differentially expressed proteins. The connection was illustrated using the web-based tool STRING.

To clarify the effect of blast exposure on the expression of specific proteins in early lung injury, lung samples were collected at 12 h, 24 h, 48 h, 72 h, and 1 week after blast exposure and analyzed by LC-MS/MS. A total of 6861 proteins were identified (Figure 2(a)). Compared with the lungs of control mice, proteins in the lungs of mice after blast exposure with fold changes ≥ 1.2 or ≤ 0.9 were subjected to the DAVID functional analysis. A total of 608 differentially expressed proteins were identified, of which 215, 128, 187, 232, and 65 proteins were identified from lung samples at 12 h, 24 h, 48 h, 72 h, and 1 w after blast exposure, respectively (Figure 2(b)). The DAVID functional analysis was used to classify these differentially expressed proteins into three categories, including cellular components (CC), biological process (BP), and molecular function (MF). At the level of BP, 130 differentially expressed proteins were associated with cell adhesion, extracellular matrix tissue, collagen fibril tissue, and coagulation function (Figure 2(c)), while analysis of CC revealed 66 differentially expressed proteins enriched in extracellular exosomes, extracellular matrix, basement membrane, and cytoplasm subunits (Figure 2(d)). Analysis according to MF revealed 43 differentially expressed proteins associated with extracellular matrix structure, actin binding, and antioxidant activity (Figure 2(e)). A total of 24 enrichment pathways were identified by the Kyoto Encyclopedia of Genes and Genomes (KEGG) enrichment analysis. The top 6 enrichment pathways included ECM-receptor interactions, focal adhesions, complement and coagulation cascades, PI3K-Akt signaling pathway, leukocyte transendothelial migration, and platelet activation (Figure 2(f)). The STRING interaction enrichment analysis was used to visualize the interaction and functional enrichment of these differentially expressed proteins at different time points (Figure 2(g)).

### 3.3. Alteration and Characteristics of Early Inflammatory Proteins in Blast-Induced Lung Injury at Different Time Points

**Figure 3:**
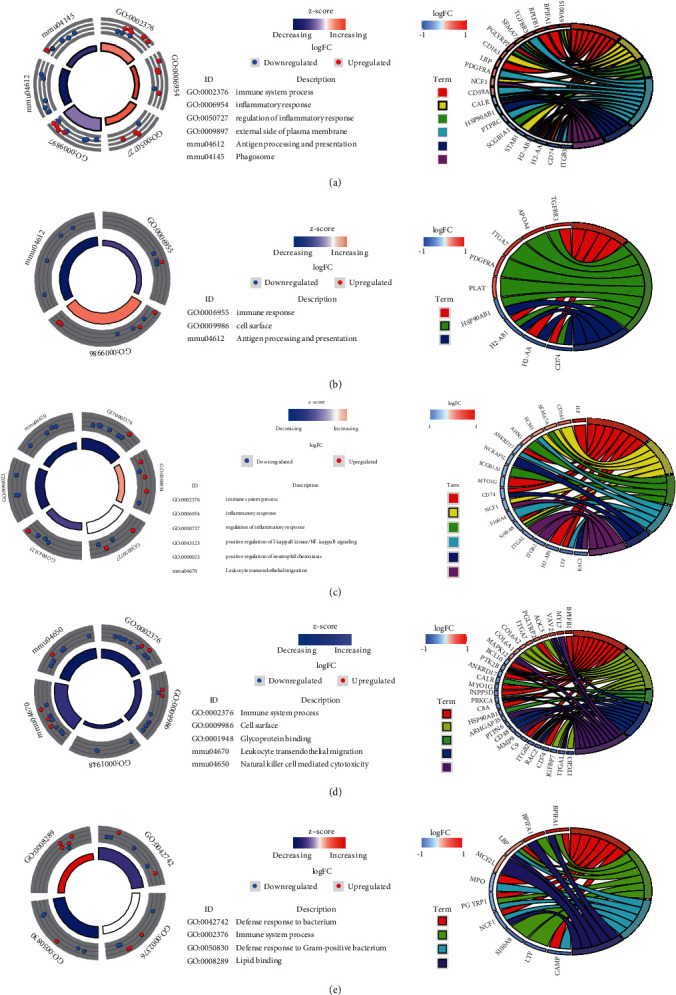
Inflammation-related differentially expressed proteins enriched in cellular components and signaling pathways at different times after blast exposure. (a) 12 h after blast exposure; (b) 24 h after blast exposure; (c) 48 h after blast exposure; (d) 72 h after blast exposure; and (e) 1 week after blast exposure. Each protein classified into cellular components and canonical pathways was marked with the same color. Each cluster of cellular components and canonical pathways in the plot is assigned a unique color. Each connection between proteins in the cellular components or canonical pathways represented the fold change.

Next, the DAVID database was further used to analyze the mouse lung tissue samples after blast exposure, and a total of 177 differentially expressed proteins were identified to be associated with lung inflammation. GO and KEGG were further used to analyze these inflammation-associated differentially expressed proteins in samples with different blast exposure times. In 12 h blast exposure samples, the differentially expressed proteins were mostly enriched in immune system processes, inflammatory response, regulation of inflammatory response, external side of plasma membrane, antigen processing and presentation, and phagosome (Figure 3(a)). In 24 h blast exposure samples, the top enriched protein domains including immune response, cell surface, antigen processing, and presentation (Figure 3(b)). In 48 h blast exposure samples, the top enriched GO terms and pathways were immune system processes, inflammatory response, inflammatory response regulation, positive regulation of I-*κ*B kinase/NF-*κ*B signaling, positive regulation of neutrophil chemotaxis, and leukocyte transendothelial migration (Figure 3(c)). In 72 h blast exposure samples, the top enriched GO terms and pathways were immune system process, cell surface, glycoprotein binding, leukocyte transendothelial migration, and natural killer cell mediated cytotoxicity (Figure 3(d)), while in 1-week blast exposure samples, the differentially expressed proteins were mostly enriched in defense response to bacterium, immune system process, defense response to Gram-positive bacterium, and lipid binding (Figure 3(e)).

### 3.4. Blast Exposure Significantly Increased the Expression of Key Proteins Involved in Inflammatory Infiltration Regulation

**Figure 4:**
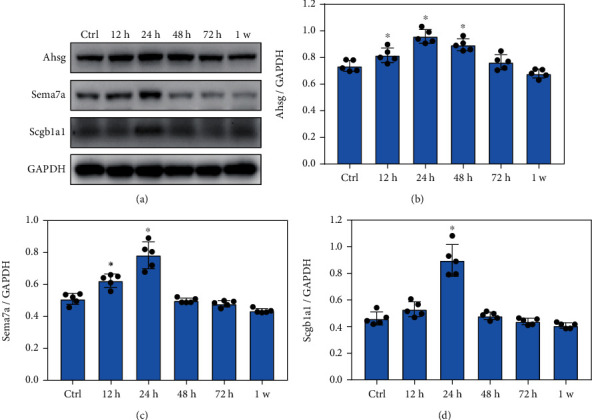
Blast exposure significantly increased the expression of key proteins involved in inflammatory infiltration regulation. (a) Western blot analysis of the expression of key proteins related to the regulation of inflammatory response after blast exposure. (b) Ahsg expression levels were significantly increased at 12 h, 24 h, and 48 h after blast exposure compared with the control (Ctrl) group. (c) Sema7a levels were significantly increased at 12 h and 24 h after blast exposure compared with the control group. (d) Scgb1a1 expressions were significantly increased at 24 h after blast exposure compared with the control group. All experiments were repeated at least three times. All data were expressed as mean ± SEM (*n* = 5) and analyzed by the one-way ANOVA test, followed by Tukey's test for multiple comparisons. Differences were considered statistically significant at *p* < 0.05 for all analyses. ^∗^*p* < 0.05 vs. the control group.

Western blotting analysis showed that Ahsg levels increased significantly at 12 h, 24 h, and 48 h after blast exposure (Figures 4(a) and 4(b), *p* < 0.05), while Sema7a levels dramatically increased at 12 h and 24 h after blast exposure (Figure 4(c), *p* < 0.05). In addition, Scgb1a1 levels obviously increased at 24 h after blast exposure (Figure 4(d), *p* < 0.05). These data suggested that blast exposure significantly alter the expression of key proteins involved in the regulation of inflammatory response.

### 3.5. Blast Exposure Significantly Increased the Expression of Key Proteins Involved in Leukocyte Transendothelial Migration

**Figure 5:**
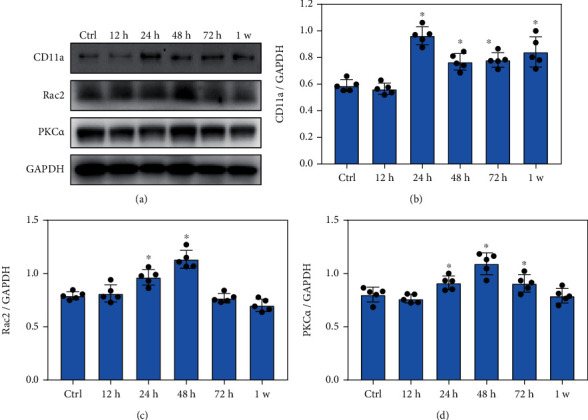
Blast exposure significantly altered the expression of key proteins involved in leukocyte transendothelial migration. (a) Western blot analysis of the expression key proteins related to leukocyte transendothelial migration after blast exposure. (b) CD11a expression levels were significantly increased at 24 h, 48 h, 72 h, and 1 week after blast exposure compared with the control (Ctrl) group. (c) Rac2 expression levels were significantly increased at 24 h and 48 h after blast exposure compared with the control group. (d) PKC*α* expressions were significantly increased at 24 h, 48 h, and 72 h after blast exposure compared with the control group. All experiments were repeated at least three times. All data were expressed as mean ± SEM (*n* = 5) and analyzed by the one-way ANOVA test, followed by Tukey's test for multiple comparisons. Differences were considered statistically significant at *p* < 0.05 for all analyses. ^∗^*p* < 0.05 vs. the control group.

Western blotting analysis showed that the levels of lymphocyte function antigen-1a (CD11a) significantly increased at 24 h, 48 h, 72 h, and 1 week after blast exposure (Figures 5(a) and 5(b), *p* < 0.05). Rac2 is a ras-related guanosine triphosphatase and mainly expressed in hematopoietic cells. Rac2 plays an important role in regulating the functions of mast cells, macrophages, and neutrophils. In this study, Rac2 levels in the lungs significantly increased at 24 h and 48 h after blast exposure (Figure 5(c), *p* < 0.05). In addition, the expression of protein kinase Ca (PKC*α*) was significantly upregulated at 24 h, 48 h, and 72 h after blast exposure (Figure 5(d), *p* < 0.05). These data suggested that blast exposure significantly altered the expression of key proteins involved in leukocyte endothelial migration during inflammatory response.

### 3.6. Blast Exposure Significantly Altered the Expression of Key Proteins Involved in the Phagocytic Pathway

**Figure 6:**
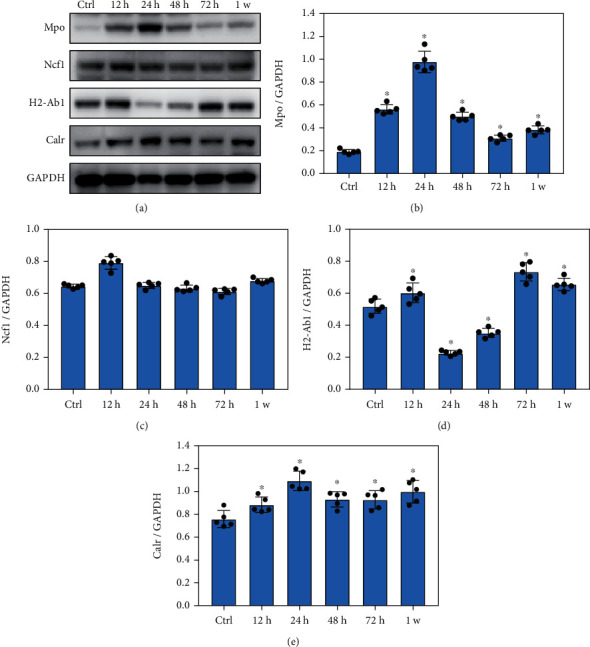
Blast exposure significantly altered the expression of key proteins in phagocytic pathway. (a) Western blot analysis of the expression of key proteins related to phagocytic pathway after blast exposure. (b) Mpo expression levels were significantly increased at 12 h, 24 h, 48 h, 72 h, and 1 week after blast exposure compared with the control (Ctrl) group. (c) Compared with control group, there was no significant difference in the Ncf1 expression at different time points after blast exposure. (d) Compared with the control group, H2-Ab1 expression levels were significantly increased at 12 h, 72 h, and 1 week but significantly decreased at 12 h and 24 h after blast exposure. (e) PKC*α* expression levels were significantly increased at 12 h, 24 h, 48 h, 72 h, and 1 week after blast exposure compared with the control group. All experiments were repeated at least three times. All data were expressed as mean ± SEM (*n* = 5) and analyzed by the one-way ANOVA test, followed by Tukey's test for multiple comparisons. Differences were considered statistically significant at *p* < 0.05 for all analyses. ^∗^*p* < 0.05 vs. the control group.

Compared with the control group, Mpo levels significantly increased at 12 h, 24 h, 48 h, 72 h, and 1 week after blast exposure (Figures 6(a) and 6(b), *p* < 0.05). However, there was no significant change in Ncf1 protein levels after blast exposure (Figure 6(c), *p* > 0.05). The H2-Ab1 protein levels increased significantly at 12 h, 72 h, and 1 week after blast exposure but decreased significantly at 24 h and 48 h after blast exposure (Figure 6(d), *p* < 0.05). PKC*α* expression was significantly increased at 12 h, 24 h, 48 h, 72 h, and 1 w after blast exposure (Figure 6(e), *p* < 0.05). These data indicated that blast exposure significantly altered the expression of critical proteins involved in the phagocytic pathway during inflammatory response.

### 3.7. Blast Exposure Significantly Changed the Expression of Crucial Proteins Involved in Immune System Process

**Figure 7:**
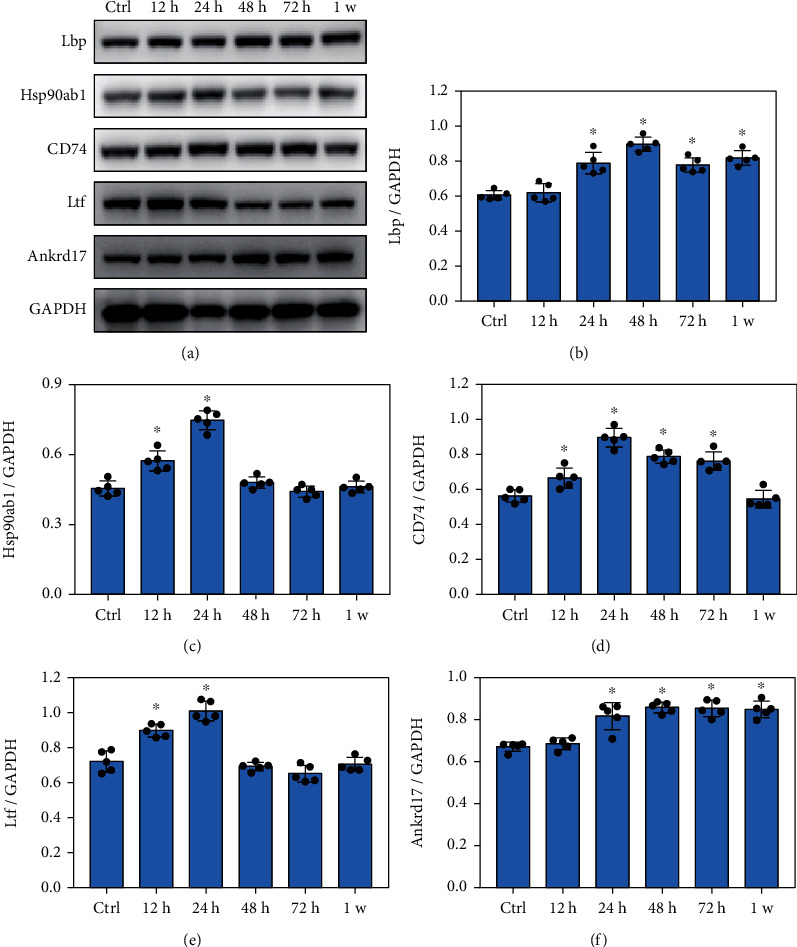
Blast exposure significantly changed the expression of crucial proteins in immune system process. (a) Western blot analysis of the expression of key proteins related to immune system process after blast exposure. (b) Lbp expression levels were significantly increased at 24 h, 48 h, 72 h, and 1 week after blast exposure compared with the control (Ctrl) group. (c) Hsp90ab1 expression levels were significantly increased at 12 h and 24 h after blast exposure compared with the control group. (d) CD74 expression levels were significantly increased at 12 h, 24 h, 48 h, and 72 h after blast exposure compared with the control group. (e) Ltf expression levels were significantly increased at 12 h and 24 h after blast exposure compared with the control group. (f) Ankrd17 expression levels were significantly increased at 24 h, 48 h, 72 h, and 1 week after blast exposure compared with the control group. All experiments were repeated at least three times. All data were expressed as mean ± SEM (*n* = 5) and analyzed by the one-way ANOVA test, followed by Tukey's test for multiple comparisons. Differences were considered statistically significant at *p* < 0.05 for all analyses. ^∗^*p* < 0.05 vs. the control group.

Compared with the control group, the expression level of Lbp significantly increased at 24 h, 48 h, 72 h, and 1 week after blast exposure (Figures 7(a) and 7(b), *p* < 0.05). Hsp90ab1 levels increased significantly at 12 h and 24 h after blast exposure (Figure 7(c), *p* < 0.05), while CD74 levels were significantly increased at 12 h, 24 h, 48 h, and 72 h after blast exposure (Figure 7(d), *p* < 0.05). Ltf levels increased significantly at 12 h and 24 h after blast exposure (Figure 7(e), *p* < 0.05), and Ankrd17 levels were significantly increased at 24 h, 48 h, 72 h, and 1 week after blast exposure (Figure 7(f), *p* < 0.05). These data suggested that blast exposure significantly alters the expression of crucial proteins involved in immune system process.

## 4. Discussion

Although inflammation is known to be an important feature of lung blast injury, the underlying molecular mechanisms of blast exposure-induced lung injury at different times remain unclear. In the present study, we first identified 6861 global proteins and 608 differentially expressed proteins in lung tissues after blast exposure, of which 215, 128, 187, 232, and 65 proteins were identified from lung samples at 12 h, 24 h, 48 h, 72 h, and 1week after blast exposure, respectively. In addition, the 177 differentially expressed proteins induced by blast exposure were associated with inflammatory responses, mainly enriched in inflammation response regulation, leukocyte transendothelial migration, phagocytosis, and immune processes. Western blotting analysis further validated the expression changes of these inflammation-related differentially expressed proteins induced by blast exposure at different times. These differentially expressed proteins provide potential targets for future drug development and provide a new perspective for the treatment of lung injury caused by blast exposure.

In recent years, blast exposure-induced inflammatory responses have received increasing attention, which may be related to the clinically observed pathological processes. A study in canine showed that blast exposure activated NF-*κ*B and MAPK signaling pathways in lung tissues and triggered nuclear translocations of NF-*κ*B, which in turn regulated the transcription of downstream genes, such as IL-6 and TNF-*α* [25]. In a phase 2 clinical trial, the use of anti-IL-1*β* antibody or blocking IL-1R expression significantly reduced the inflammatory response caused by blast exposure and significantly improved the symptoms of patients with severe blast injuries [26]. The lung began to exhibit early inflammatory after 3 h of blast exposure, followed by epithelial cell damage after 12 h of blast exposure, and the inflammation and oxidation reached the peak at 24 h and 48 h [17]. The results of the present study demonstrated that blast exposure not only caused significant hemorrhage, edema, and inflammatory cell infiltration but also greatly increased the expression of TNF-*α*, IL-1*β*, and TGF*β*1 and reduced the expression of IL-10 in the lungs. It has been reported that blast exposure led to an early inflammatory response, accompanied by significant oxidative stress and apoptosis in lung tissue cells [16]. A similar study also found that blast exposure significantly increased the expression of systemic proinflammatory and anti-inflammatory cytokines and significantly caused pulmonary edema, inflammation, endothelial damage, and bleeding. Moreover, the administration of decay accelerating factor effectively inhibited the inflammatory response by regulating the complement system [27]. Another study by Zhang et al. found that blast exposure obviously reduced cell adhesion abilities of A549 cells in vitro and induced inflammatory response, apoptosis, and oxidative damage [28]. Perfluorocarbon administration significantly inhibited blast-induced inflammation through NF-*κ*B and MAPK signaling pathways. Chen et al. found that after blast injury, early peritoneal dialysis can significantly reduce pulmonary edema, inflammation, and the expression of IL-1*β*, IL-6, TNF-*α*, monocyte chemoattractant protein-1 (MCP-1), and C-reactive protein [9]. These evidence also support our findings that blast exposure can cause severe lung injury, lung leukocyte infiltration, and the production of inflammatory factors in mice.

To explore the underlying molecular mechanism of lung blast injury at different times, LC-MS/MS analysis was conducted to analyze the differential expression profiles of mouse lung samples collected at 12 h, 24 h, 48 h, 72 h, and 1 week after blast exposure. We identified 6861 global proteins and 608 differentially expressed proteins through lung samples at different time points after blast exposure. Among them, 177 differentially expressed proteins were enriched in the inflammation response regulation, leukocyte transendothelial migration, phagocytosis, and immune processes. These findings are supported by another report [29] that inflammation caused by blast injury was closely related to immune regulation, and blast exposure caused corneal edema, neovascularization, and immune cell infiltration. In other words, this indicated that corneal injury may be related to the activation of the immune system and the infiltration of lymphocyte. In addition, data from mouse retinal microarrays after blast injury showed that biological processes include loss of synaptic transmission, impaired cell metabolism, and activation of the immune system [30]. Most genes upregulated in the KEGG pathway were related to the activation of inflammation. A previous study revealed that blunt chest trauma caused local and systemic inflammatory changes in mice, and alveolar macrophage can significantly improve the posttraumatic inflammatory responses [31]. In this study, Ahsg, Sema7a, and Scgb1a1, the key proteins that regulate the inflammatory response to blast injury, were significantly upregulated at 12 h and 48 h after blast exposure. Ahsg is a cysteine protease inhibitor secreted by the liver, which can inhibit vascular calcification by preventing spontaneous mineral deposition in blood vessels [32]. Ahsg was a negative acute-phase reaction protein, mediated by a variety of proinflammatory mediators including TNF-*α*, thereby reducing the inflammatory response to injury and infection [33, 34]. Moreover, reduced serum Ahsg levels in patients with mild to moderate Alzheimer's disease were associated with cognitive impairment and high levels of TNF-*α* [35]. On the other hand, Sema7a can induce the production of proinflammatory cytokines in endothelial cells and epithelial cells and promote the migration of neutrophil from vascular endothelium. Moreover, Sema7a knockout reduced the lipopolysaccharide-treated inflammatory response in the lungs [36]. In the study of acute lung injury caused by seawater aspiration [37], Sema7a contributed to seawater-induced pulmonary edema and inflammation via the Plexin C1/*β*1 integrin pathway. Blockage with the plexin C1 antibody inhibited endothelial cytoskeleton remodeling and endothelial permeability in rats, and silencing Sema7a by siRNA inhibited VEGF expression. Combining our finding and these studies confirms that blast exposure significantly changes the expression of key proteins involved in the regulation of inflammatory response.

Although leukocytes play a key role in the host defense against bacterial infection, the excessive accumulation of leukocytes in the lung microcirculation may affect gas exchange in the lungs. Furthermore, leukocyte aggregation is the rate-limiting step in acute inflammatory lung injury. In this study, it was found that blast exposure significantly increased the expression of key proteins involved in leukocyte endothelial migration, including CD11a, Rac2, and PKC*α*. CD11a is an important T cell integrin and plays an important role in regulating T cell activation and migration. Integrins are crucial receptor on the T cells surface that regulate cell adhesion, signal transduction, and migration. CD11a can cooperate with adhesion molecule ICAM-1 to increase the adhesion capacity of endothelial cells, prolong the contact time with antigen-presenting cells, or strengthen the binding of target cells for cell killing [38]. The results of studies of endotoxemia and sepsis animal models showed that leukocyte adhesion capacity was mediated by the *β*2-integrin family, and targeting CD11a/CD18 reduced leukocyte infiltration in the lungs. In addition, CD11a and CD11b were found to mediate leukocyte adhesion in the arterioles and venules, and CD11a was considered to support leukocyte rolling in the pulmonary arterioles, suggesting that leukocyte rolling depends on CD11a in the lungs [39]. Leukocyte motility is necessary for host defense response, and Rac-family is involved in leukocyte defense function. Rho small GTPase Rac2 is functionally involved in many important neutrophil capacities. Rac2 gene knockout resulted in insufficient basic motor function of neutrophils and macrophages in larval zebrafish, which in turn reduced the ability to response to local wounds or infections [40]. Study by Tell et al. also found that loss of Rac2 activity led to neutrophil dysfunction and subsequent severe bacterial infection [41]. These data suggest that blast exposure significantly modulates the expression of key proteins involved in leukocyte endothelial migration during inflammatory response.

Phagocytosis is a very important part of the inflammation process. Neutrophils or monocytes can migrate from blood vessels to the lesion site under the induction of chemokine and remove necrotic tissue by phagocytosis. Myeloperoxidase (Mpo), H2-Ab1, and PKC*α* were closely related to cell phagocytosis during blast exposure-induced inflammation. In this study, we showed that blast exposure significantly increased the expression levels of Mpo, H2-Ab1, and PKC*α* at different time points. The heme-rich granzyme Mpo of neutrophils and monocytes has the potential to regulate subcellular signaling, immune response, microvascular hemostasis, and ECM remodeling. Targeting Mpo can reduce oxidative damage to host tissue and subsequent inflammatory response. It was reported that the levels of IL-1*β* and Mpo in feces of patients with ulcerative colitis were positively correlated with the severity of the disease [42]. Neutrophils and monocytes are known to be the essential innate immunity members for the removal of pathogens and damage and are also signs of acute inflammation. In addition, the cytokines they produce can recruit other immune cells and regulate the adaptive immune response by affecting the activity of antigen presenting cells. Neutrophils and monocytes also produce harmful amounts of reactive oxygen species through the Mpo pathway as part of the phagocytic/biological killing function. Neutrophils are a main production source of Mpo, which is mainly recruited by IL-8 chemotaxis. In addition, H2-Ab1 is an important molecule in antigen-presenting cells and lymphocytes. H2-Ab1 expression was upregulated by sleep deprivation and melatonin treatment and may be involved in sleep deprivation and melatonin-mediated colonic inflammation [43]. In the 2,3,7,8-tetrachlorodibenzo-p-dioxin- (TCDD-) induced inflammation, levels of several major histocompatibility complex (MHC) class II genes such as H2-Aa, H2-Ab1, H2-DMb1, and Cd74 were inhibited in TCDD-treated mice [44], which is consistent with the decrease in the levels of macrophages and dendritic cells in the lamina propria, and indicate that antigen-presenting cells migrate out of the intestine. These data indicate that blast exposure alters the expression of critical proteins involved in the phagocytic pathway during inflammatory response.

The inflammatory response caused by blast injury is closely related to immune regulation. It has been found that lung blast injury led to corneal edema, neovascularization, and immune cell infiltration, which indicated that corneal injury may be related to immune system activation and lymphocyte infiltration [29]. Data from retinal microarray in blast-injured mice also showed that the main biological processes related to GO gene enrichment were loss of synaptic transmission, impairment of cell metabolism, and activation of immune system. This indicates that half of the upregulated genes after KEGG pathway classification were functionally associated to immune system activation, and this acute activation was consistent with the increase of acquired immune system markers. On the other hand, T lymphocyte markers CD3 and CD8 in blast-injured mice were significantly increased, and the aggregation and activation of T cells suggested that cellular immune mechanism might be involved in the repair process of blast-induced retinal injury [30]. After blast lung injury, CD43Lo/His48Hi monocytes in blood and bronchoalveolar lavage fluid of rats showed a strong selective response, accompanied by a significant increase in spleen mononuclear macrophages. This suggests that the distribution of immune cells in blood and lung tissue can provide a new research direction for monitoring, evaluating, or weakening the immune response of patients with blast injury [45]. In this study, we found that blast exposure significantly changed the expression of crucial proteins involved in immune system process, including Lbp, Hsp90ab1, CD74, Ltf, and Ankrd17. Lbp is an important secretory acute-phase protein in response to bacterial infection. Lbp can act as a lipid transfer protein to transfer LPS to the CD14/TLR4 receptor complex on immune cells, thereby enhancing the immune response to LPS [46]. It has been reported that high levels of Lbp in the serum of septic patients attenuated the inflammatory response of macrophages and monocytes to LPS [47]. In addition to systemic infection, infectious and allergic lung inflammation can also lead to increased serum Lbp levels [48]. In addition, Lbp can remove the LPS bound by CD14 and MD-2 on the cell membrane, thereby inhibiting inflammatory response. This indicates that Lbp actively can inhibit LPS-mediated transfer of CD14/TLR4/MD-2 receptors to the proinflammatory pathway [49]. On the other hand, Lbp can be considered an important regulator of the pulmonary immune response. Lbp can respond to small amounts of LPS through a self-limited inflammatory response, which may help to neutralize higher doses of LPS to prevent the harmful consequences of exaggerated lung inflammation [50]. Similarly, in this study, Lbp expression levels increased significantly at 24 h, 48 h, 72 h, and 1 week after blast exposure. As to CD74, macrophage migration inhibitory factor (MIF) can bind to CD74 on the cell surface, leading to phosphorylation of CD74 cytoplasmic domains and membrane recruitment of CD44. The MIF-CD74-CD44 complex can subsequently activate members of nonreceptor tyrosine kinases of Src family to initiate signal transduction. CD74 also play an important role in of MHC class II antigen presentation in autoimmune diseases, such as systemic lupus erythematosus [51]. In addition to autoimmune disease, CD74 plays a role in renal tubular epithelial cells. CD74 deficiency not only reduced lupus-like autoimmunity but also reduced renal pathology in chronic graft-versus-host mice [52]. In addition, CD74 knockout also reduced the surface expression of MHC class II and severely attenuated Th2 response, but the Th1 response was relatively preserved. Similar to CD74 knockout mice, MIF knockout mice also showed Th2 cytokine deficiency, which may be related to the signal transmission of MIF/CD74 complex or impaired cell survival [53]. These data suggest that blast exposure significantly changed the expression of crucial proteins involved in immune system process.

In conclusion, our data suggested that early inflammatory response may be the initiating factor for lung blast injury. Blast exposure induced early inflammatory response in lung tissues by regulating the expression of key proteins in the inflammatory process. In addition, these data further provided candidate targets/approaches for the future development of lung blast injury therapy.

## Data Availability

All authors declare that all data are fully available without restriction.
